# Supplementary material for: The Reasons for Unfinished Nursing Care during the COVID-19 Pandemic: An Integrative Review
Source: Nurs Rep. 2024 Mar 27;14(2):753–66. doi: 10.3390/nursrep14020058 (PMC11036286; doi:10.3390/nursrep14020058)
Supplement: Supplementary file 1 [file nursrep-14-00058-s001.zip › nursrep-2864938-supplementary.pdf]

Supplementary Materials Table S1: Search strategies used in approached databases

Database and search strategy	Results obtained
**PubMed**<br>("unfinished nursing care "[All Fields] OR " Rationing Nursing care "[All Fields] OR " missed nursing care "[All Fields]) AND ("reasons "[All Fields] OR "causes" OR "factors/predictors)	172
**CINAHL**<br>("unfinished nursing care "OR " Rationing Nursing care " OR " missed nursing care ") AND ("reasons " OR "causes" OR factors/predictors")	228
**Scopus**<br>("unfinished nursing care "OR " Rationing Nursing care " OR " missed nursing care ") AND ("reasons " OR "causes" OR factors/predictors")	34

Legend: CINAHL The Cumulative Index to Nursing and Allied Health Literature

Supplementary Materials Table S2: Study quality Assessment: Critical Appraisal Skills Programme (CASP) for a Qualitative Research (Critical Appraisal Skills Programme; CASP 2018) [18]

	**Chiappinotto & Palese, 2022 [20]**	**Chiappinotto et al., 2023 [21]**	**Safdari et al., 2023 [22]**
**Item 1.** Was there a clear statement of the aims of the research?	Y	Y	Y
**Item 2.** Is a qualitative methodology appropriate?	Y	Y	Y
**Item 3.** Was the research design appropriate to address the aims of the research?	CT	Y	Y
**Item 4**. Was the recruitment strategy appropriate to the aims of the research?	Y	Y	Y
**Item 5.** Was the data collected in a way that addressed the research issue?	Y	Y	Y
**Item 6.** Has the relationship between researcher and participants been adequately considered?	Y	Y	Y
**Item 7.** Have ethical issues been taken into consideration?	CT	Y	Y
**Item 8.** Was the data analysis sufficiently rigorous?	Y	Y	Y
**Item 9.** Is there a clear statement of findings?	Y	Y	Y
**Item 10.** How valuable is the research?	Y	Y	Y

Legend*:* Y: Yes; N: No; CT: Can't tell

Supplementary Materials Table S3: Study quality Assessment: Mixed-Method Appraisal Tool (MMAT) [19]

**Hackman et al., 2023 [23]**			
**Item 1.** Are there clear research questions?	Y	**Item 10** Are the findings adequately derived from the data?	Y
**Item 2.** Do the collected data allow to address the research questions?	Y	**Item 11** Is the interpretation of results sufficiently substantiated by data?	Y
**Item 3.** Is there an adequate rationale for using a mixed methods design to address the research question?	N	**Item12** Is their coherence between qualitative data sources, collection, analysis and interpretation?	Y
**Item 4.** Are the different components of the study effectively integrated to answer the research question?	N	**Item 13** Is the sampling strategy relevant to address the research question?	Y
**Item 5.** Are the outputs of the integration of qualitative and quantitative components adequately interpreted?	Y	**Item 14** Is the sample representative of the target population?	Y
**Item 6.** Are divergences and inconsistencies between quantitative and qualitative results adequately addressed?	Y	**Item 15** Are the measurements appropriate?	Y
**Item 7.** Do the different components of the study adhere to the quality criteria of each tradition of the methods involved?	Y	**Item 16** Is the risk of nonresponse bias low?	N
**Item 8.** Is the qualitative approach appropriate to answer the research question?	Y	**Item 17** Is the statistical analysis appropriate to answer the research question?	Y
**Item 9.** Are the qualitative data collection methods adequate to address the research question?	CT	**-**	-

Legend*:* Y: Yes; N: No; CT, Can't Tell

Supplementary Materials Table S4: Description of included studies

**Authors, year, country, context and study period**	**Objective, type of study, data collection process**	**Sampling methodology, Participants, Demographic data**	**Results**
Chiappinotto & Palese, 2022 [20]<br>Italy<br>A large public health care trust of the National Health Service, Northeast Italy with 9332 health workers, including 3868 nurses, and organized in six hospitals with a total of 2390 beds.<br>Period: May to August 2021	To investigate the reasons for UNC at all levels of nursing service<br><br>Qualitative study<br><br>Semi-structured online and face-to-face interviews	Convenience<br>nurses (n=29): clinicians (n=19)<br>nurse managers (n=7), managers (n= 3)<br>Women: 27/29<br>Age in years (mean): 35.6 nurses, 48.1 coordinators, 50 managers<br>Role experience in years (mean): 11.2 nurses, 4.3 coordinators, 7.7 managers	UNC reasons identified on five levels:<br>(1) System: "Insufficient nursing support".<br>(2) Unit: "Inadequate care environment", "Inadequate material resources", "Inadequate human resources", "Ineffective intra-professional collaboration", "Ineffective work processes", "Ineffective shift planning" and "Ineffective nursing care delivery models";<br>(3) Nurse manager level: "Inadequate coordinator leadership";<br>(4) Nurse level: "Ineffective performance of clinical nurses", "Deficiencies in training", "Inadequate humanistic view of the patient" and "Ineffective prioritisation skills";<br>(5) Patient: "Increased demand for patient care" and "Lack of caregiver support".
Chiappinotto et al., 2023 [21]<br>Italy<br>Two medical wards (66 beds each) two surgical wards (52 beds each) of a large discharge hospital (35,000 admissions/year)<br>Period: April to June 2022	To explore the reasons for UNC perceived by patients.<br><br>Qualitative study<br>Semi-structured interviews	Convenience<br>Patients: 23<br>Women: 11/23<br>Age in years: 66.2 mean<br>Experience in the Hospital care: hospitalized for more than 48 hours	UNC factors articulated in four levels:<br>(1) System: "New health system priorities" and '"Pre-existing fragility of health structures";<br>(2) Unit: "Lack of resources allocated to operational units", "Ineffective organisation of operational units" and "Inadequate leadership of the coordinating nurse";<br>(3) Nurses: "Attitudes and competences of the nursing staff";<br>(4) Patient: "Increased care needs and expectations"
Safdari et al., 2023 [22]<br>Iran<br>Three hospitals considered as referral centres for patients with COVID-19.<br>Period: December 2020 to February 2021	To investigate factors influencing missed care during COVID-19 from the nurses' point of view<br><br>Qualitative study<br>Semi-structured interviews	Intentional<br>Nurses: 14<br>Age in year: 31.85 mean<br>Role experience in years (mean): 7.7	Reasons/factors categorised in four main categories:<br>(a) Care-related factors, e.g., uncertainties in care;<br>(b) Disease-related factors, e.g., extent of symptoms;<br>(c) Patient-related factors, e.g., comorbidity, elderly patients; and<br>(d) Organisational related factors, e.g., lack of human resources, unfavourable working environment.
Hackman et al., 2023 [23]<br>Finland<br>Nursing Home<br>Period: January to May 2021	To describe uncompleted nursing care activities in residences for the elderly and the reasons for UNC<br><br>Cross-sectional study<br>Online BERNCA-NH questionnaire with a final open question	Convenience<br>Health workers: 468 out of 2700 (17.8%)<br>Women: 462 (95.1%)<br>Age 35-55: 249 (51.3%)<br>Role experience in years (mean):14	On 7.3 out of 20 nursing activities remained unfinished: the most frequently unfinished nursing activities were: cultural and social for residents, creation of residents' care plans.<br>Five main categories of reasons leading to UNCs:<br>(a) Insufficient resources, e.g., lack of human resources, lack of expertise;<br>(b) Patient characteristics, e.g., health status;<br>(c) Unexpected situations in work units, which may be internal (e.g., consulting paramedics or physicians) or external (e.g. cold weather);<br>(d) Lack of cooperation, e.g., non-nursing activities and administrative activities;<br>(e) Challenges in organising and directing work, e.g., lack of a functioning work team.

Abbreviations: UNC: Unfinished Nursing Care; N/A: not applicable, BERNCA-NH: Basel extent of rationing of nursing care for nursing homes instrument

Supplementary Materials Table S5: Study Limitations

Limitations	Integrative review
Including Publication Bias	Have introduced some selection bias
Time-Lag Bias	*The publication time lag (we included studies conducted during the pandemic, and more may be in the process of being published)
Language Bias	*The language limitations
Outcome reporting Bias	Moreover, some studies have investigated reasons with different methodologies, sometimes as predictors/factors and other times as experiences. We used the concepts interchangeably, even if they have different meanings as reasons associated with the UNC phenomenon and factors as influencing the occurrence of UNC. In the future, it will be necessary to reflect, from a methodological point of view, on their different meaning

Abbreviations: UNC: Unfinished Nursing Care
